# The Association Between Ferritin and Vitamin D Levels in Premenopausal Fibroid Uterus Cases With Anemia

**DOI:** 10.7759/cureus.13392

**Published:** 2021-02-17

**Authors:** Suchitra Kumari, Pavuluri Swetha, Shyam Krishnan R, Saurav Nayak, Sweta Singh

**Affiliations:** 1 Biochemistry, All India Institute of Medical Sciences, Bhubaneshwar, IND; 2 Biochemistry, All India Institute of Medical Sciences, Bhubaneswar, IND; 3 Obstetrics and Gynaecology, All India Institute of Medical Sciences, Bhubaneswar, IND

**Keywords:** fibroid uterus, anaemia, ferritin, vitamin d deficiency

## Abstract

Objective

The present study aimed to evaluate the association between serum ferritin and vitamin D levels in fibroid uterus cases presenting with anemia.

Methods

Sixty premenopausal women with uterine fibroids (30 associated with anemia and 30 without anemia) were enrolled as cases and control. All participants were evaluated on the basis of a questionnaire, which included queries related to obstetric, medical, and sociodemographic history. Peripheral blood smear, complete blood count (CBC), hemoglobin (Hb), and serum ferritin concentration were measured by a fully automated analyzer, and 25(OH) vitamin D level was measured by enzyme-linked immunosorbent assay (ELISA).

Results

There was a significant difference in ferritin levels between cases and control (p<0.001). The exposure to sunlight was moderate (one-hour exposure) in all subjects, eliminating the confounding effect of sunlight exposure influencing vitamin D levels. The median vitamin D level in cases was 5.0 ng/ml [interquartile range (IQR): 4.8], and that in control was 18.4 ng/ml (IQR: 7.9; p<0.001). A strong positive correlation of (r)=0.616 (p<0.001) was found between serum ferritin and vitamin D levels.

Conclusion

Fibroid uterus cases with anemia are more prone to vitamin D deficiency as compared to cases without anemia. Vitamin D estimation in fibroid uterus cases presenting with anemia would be useful for better patient management.

## Introduction

Uterine fibroid (leiomyoma) is the most common benign tumor in the uterus, and there is a growing prevalence of fibroid uterus in India [1], which has been associated with major health and economic burden in women of reproductive age group. Fibroid uterus occurs in 20-40% of women of reproductive age, and one-third of gynecology admissions happen to be fibroids with menorrhagia, anemia, and lump in the abdomen with pain [2] severe enough to significantly impact women’s quality of life. Submucosal fibroids cause severe bleeding per vagina followed by intramural type resulting in anemia [3]. Studies have highlighted vitamin D deficiency as a major risk factor for the development of uterine fibroids [4,5]. Though factors like race, ethnicity, tradition, culture, seasonal variation, exposure to sunlight, and nutritional status play a major role in regulating vitamin D levels, the cause of hypovitaminosis D in fibroids is still a topic of active research. Recently, the co-existence of anemia and hypovitaminosis D has emerged as a new causal association [6] in fibroid uterus cases, but many studies have also reported vitamin D deficiency without anemia [7,8]. Vitamin D influences iron metabolism and erythropoiesis, whereas iron is essential for vitamin D synthesis as well [9]. In terms of anemia, some studies have reported that ferritin levels are positively associated with serum vitamin D [10-12]. Though the existing scientific data indicates the role of vitamin D in fibroid uterus cases, the co-existence of anemia with vitamin D deficiency and the association between ferritin and vitamin D levels in premenopausal women with fibroid uterus are still not fully understood. The present study was conducted to estimate serum ferritin, vitamin D, and hemoglobin (Hb) levels in fibroid uterus cases presenting with anemia and to compare these values with premenopausal fibroid uterus cases presenting without anemia. Further evaluation was done to determine the association between ferritin and serum vitamin D levels in anemic and non-anemic fibroid uterus women to ascertain the correlation.

## Materials and methods

A hospital-based case-control study was conducted in the Department of Biochemistry, All India Institute of Medical Sciences, Bhubaneswar after obtaining approval from the Institute Ethical Committee. Sixty clinically diagnosed premenopausal women with uterine fibroids (30 cases with anemia and 30 cases without anemia) within the age group of 25-45 years, irrespective of their parity, who were attending the outpatient section of the Obstetrics and Gynaecology Department, and who satisfied the inclusion criteria, were enrolled randomly in this study. Post-menopausal women, women with inflammatory disorders, acute infections, and chronic liver and kidney diseases, and those with autoimmune conditions were excluded from the study.

All participants were evaluated on the basis of a questionnaire prepared pertaining to the study, which included queries related to parity, age at menarche, last menstrual period, breastfeeding, type of delivery, history of intake of oral contraceptive pills (OCPs), hormone replacement therapy, miscarriage, duration of exposure to sunlight (<1 hour/day, one hour/day, >1 hour/day), race, ethnicity, history of chronic diseases like diabetes, hypertension, renal disorders, inflammatory conditions, autoimmune disorders, history of recent blood transfusion, history of intake of multivitamin/iron supplements, and the type of nutrition. The blood pressure of the participants was recorded, and height and weight were measured to calculate their body mass index (BMI).

Biochemical analysis

After obtaining informed written consent, a 5-ml blood sample was collected from all participants. The serum was stored at -20 °C until it was used for biochemical evaluation. Peripheral blood smear, complete blood count (CBC), and Hb concentration were measured by a fully automated analyzer (AU platform) using system-compatible kits. The serum ferritin levels were estimated by three-site sandwich immunoassay using direct chemiluminometric technology in chemiluminescence immunoassay analyzer (ADVIA Centaur®, Siemens Healthineers, Erlangen, Germany). Serum vitamin D levels were estimated by enzyme-linked immunosorbent assay (ELISA) method (sandwich ELISA) using a commercial 25(OH) vitamin D ELISA Kit (Elabscience, Houston, TX) as per the manufacturer's recommendations.

Based on the data obtained from the questionnaire as well as the biochemical analysis, the patients were divided into two groups: group A: fibroid with anemia (treated as cases); group B: fibroid without anemia (treated as the control group). Subjects in the anemic group (group A) were further classified into iron deficiency anemia (Hb: <11g/dl, ferritin: <12ng/ml) and iron deficiency state (Hb: >11g/dl, ferritin: <12ng/ml). Subjects in the non-anemic group (group B) were those with Hb of >11 g/dl and ferritin of >12 ng/ml. Vitamin D status analyzed by estimating 25(OH) vitamin D levels (circulating active metabolite) in the serum was categorized as vitamin D deficiency, with 25(OH)D levels of <20ng/ml; vitamin D insufficiency, with 25(OH)D levels of 20-30 ng/ml; and vitamin D sufficiency, with 25(OH)D levels of >30 ng/ml.

Statistical analysis

The data were analyzed using the SPSS Statistics software (IBM, Armonk, NY). The Mann-Whitney U test was used for comparing the two groups. We investigated the Pearson’s correlations between the hematologic parameters, i.e., ferritin and 25(OH)D levels. The odds ratio was calculated to determine the strength of association between the variables and uterine fibroids. To evaluate the odds ratio between iron deficiency anemia, iron deficiency state, and vitamin D deficiency/insufficiency subjects, the iron deficiency anemia and iron deficiency state groups, as well as the vitamin D deficiency and vitamin D insufficiency groups, were combined to form two separate groups for further analysis. Multivariate logistic regression analysis was also performed.

## Results

The clinical characteristics of the study groups were evaluated (Table 1). The data was found to be non-parametric in the Kolmogorov-Smirnov test. The median ages of fibroid subjects with anemia and those without anemia were more or less the same. The median BMI of the anemic group was 22.7 kg/m^2^ [interquartile range (IQR): 3.6], and that of the control group was 20.3 kg/m^2^ (IQR-4.7, p=0.008). There was a significant difference in Hb levels between the two groups. The mean corpuscular volume (MCV), mean corpuscular Hb (MCH), and mean corpuscular Hb concentration (MCHC) also showed a significant difference between the groups (p<0.001 for each), so did the packed cell volume (PCV) (p<0.001) and red cell distribution width (p=0.037). There was no significant difference in RBC counts between cases and control (p=0.717). The median ferritin level in cases was 8.0 ng/ml (IQR: 4.1), and that in control was 47.1 ng/ml (IQR: 59), and the difference was found to be statistically significant (p<0.001). The median vitamin-D level in fibroid cases with anemia was 5.0 ng/ml (IQR: 4.8), and that in cases without anemia was 18.4 ng/ml (IQR-7.9), which also indicated a significant difference (p<0.001).

**Table 1:** Clinical characteristics of the study population Data was found to be non-parametric in the Kolmogorov-Smirnov test. P-values were calculated using the Mann-Whitney U test *Significant p-value (<0.05) IQR: interquartile range; BMI: body mass index; SBP: systolic blood pressure; DBP: diastolic blood pressure; MCV: mean corpuscular volume; MCH: mean corpuscular hemoglobin; MCHC: mean corpuscular hemoglobin concentration; RBC: red blood cells; WBC: white blood cells; PCV: packed cell volume; RDW: red cell distribution width

Parameters	Fibroid subjects without anemia, median (IQR)	Fibroid subjects with anemia, median (IQR)	P-value
Age, years	42.5 (10.5)	44.0 (7.5)	0.736
BMI, kg/m^2^	20.3 (4.7)	22.7 (3.6)	0.008*
SBP, mmHg	110.5 (10)	113.0 (10)	0.804
DBP, mmHg	76.0 (10)	73.0 (10)	1.000
Hemoglobin, g/dl	12.5 (0.8)	9.8 (2.0)	<0.001*
MCV, fl/cell	86.5 (6.1)	75.0 (10)	<0.001*
MCH, pg/cell	28.2 (1.9)	22.5 (5.2)	<0.001*
MCHC, g/dl	31.7 (1.6)	30.2 (1.5)	<0.001*
RBC, million/mm^3^	4.5 (0.9)	4.4 (1.1)	0.717
WBC, thousand cells/mm^3^	7.1 (2.7)	7.6 (2.3)	0.762
Platelets, lakhs/mm^3^	2.8 (1.0)	3.3 (1.4)	0.070
PCV, %	38.1 (7.1)	32.3 (6.3)	<0.001*
RDW, %	15.2 (5.8)	17.2 (3.0)	0.037*
Urea, mg/dl	16.5 (5.0)	18.0 (4.0)	0.427
Creatinine, mg/dl	0.7 (0.2)	0.7 (0.2)	0.694
Ferritin, ng/ml	47.1 (59.0)	8.0 (4.1)	<0.001*
Vitamin D (ng/ml)	18.4 (7.9)	5.0 (4.8)	<0.001*

Table 2 compares the baseline characteristics of the two groups. The comparison was done using the chi-square test. The number of fibroids or type of fibroids was more or less the same in the two groups. Obstetric parameters such as parity, history of breastfeeding, and the use of oral OCPs did not differ much between cases and controls; 70% of the cases had a microscopic hypochromic appearance in the peripheral smear, which was less for controls. Only nine (30%) had a normocytic normochromic appearance in the anemic group, but 28 (93.3%) subjects in the control group had a normal appearance. This difference was significant (p<0.001). The exposure to sunlight was moderate (one-hour exposure) in all subjects, thereby eliminating the confounding effect of sunlight exposure influencing vitamin D levels in fibroid uterus cases.

**Table 2:** Baseline characteristics of the study population *Significant p-value (<0.05) (calculated by chi-square test) DM: diabetes mellitus

Parameters		Fibroid subjects without anemia, n (%)	Fibroid subjects with anemia, n (%)	P-value
Associated type 2 DM		3 (10)	3 (10)	1.000
Associated hypertension		0 (0)	3 (10)	0.237
Associated thyroid disorder		6 (20)	4 (13.3)	0.551
Number of fibroids	1	23 (76.7)	17 (56.7)	0.104
	2	3 (10)	1 (0.3)	
	3	0 (0)	1 (0.3)	
	Multiple	4 (13.3)	11 (36.7)	
Type of fibroid	Submucosal	7 (23.3)	7 (23.3)	0.072
	Intramural	13 (43.3)	20 (66.7)	
	Subserosal	10 (30.3)	3 (10)	
Size of fibroid	<7 cm	17 (56.7)	18 (60)	0.739
	>7 cm	13 (43.3)	12 (40)	
Menarche	<13 years	6 (20)	11 (36.7)	0.252
	>13 years	24 (80)	19 (63.3)	
Parity	Nulliparous	8 (26.7)	4 (13.3)	0.290
	Uniparous	5 (16.7)	9 (30)	
	Multiparous	17 (56.7)	17 (56.7)	
History of breastfeeding	Yes	21 (70)	26 (86.7)	0.209
	No	9 (30)	4 (13.3)	
History of miscarriage	Yes	8 (26.7)	6 (20)	0.761
	No	22 (73.3)	24 (80)	
History of oral contraceptive pill use	Yes	1 (3.3)	2 (6.7)	1.000
	No	29 (96.7)	28 (93.3)	
Peripheral smear	Microcytic hypochromic	2 (6.7)	21 (70)	<0.001*
	Normocytic normochromic	28 (93.3)	9 (30)	
Exposure to sunlight	<1 hour/day, 1 hour/day, >1 hour/day	0 (0), 30 (100), 0 (0)	0 (0), 30 (100), 0 (0)	0.639

Table 3 compares the characteristics of the study population between the groups according to their vitamin D concentration. The Kruskal-Wallis test was used for comparison. In the anemic group, 28 subjects were found to be with vitamin D deficiency with two participants having vitamin D insufficiency. In the control group, the distribution was 19 subjects with vitamin D deficiency, nine with vitamin D insufficiency, and two with vitamin D sufficiency. There was no significance between most of the parameters according to vitamin D levels. But serum ferritin showed a significant difference (p=0.001) between the three subgroups based on vitamin D levels in the control group. Among the cases, serum ferritin showed a significant difference (p=0.007) between the vitamin D subgroups.

**Table 3:** Characteristics of the study population based on serum vitamin D concentrations between the groups *Significant p-value (<0.05) (calculated using the Kruskal-Wallis test) SD: standard deviation; BMI: body mass index; SBP: systolic blood pressure; DBP: diastolic blood pressure; MCV: mean corpuscular volume; MCH: mean corpuscular hemoglobin; MCHC: mean corpuscular hemoglobin concentration; RBC: red blood cells; WBC: white blood cells; PCV: packed cell volume; RDW: red cell distribution width

Parameters	Fibroid subjects without anemia, mean ± SD				Fibroid subjects with anemia, mean ± SD		
	Vitamin D deficiency (<20 ng/ml)	Vitamin D insufficiency (20-30 ng/ml)	Vitamin D sufficiency (>30 ng/ml)	P-value	Vitamin D deficiency (<20 ng/ml)	Vitamin D insufficiency (20-30 ng/ml)	P-value
Age, years	41.6 ± 6.7	40.0 ± 8.2	42.5 ± 2.1	0.992	41.9 ± 6.3	44.0 ± 0.7	0.737
BMI, kg/m^2^	20.1 ± 3.6	20.4 ± 4.8	22.0 ± 4.4	0.823	23.1 ± 3.7	23.7 ± 3.1	0.868
SBP, mmHg	113.0 ± 9.8	117.2 ± 10.3	116.0 ± 2.8	0.548	114.9 ± 9.1	115.0 ± 21.2	0.899
DBP, mmHg	75.7 ± 8.4	74.8 ± 7.0	67.0 ± 4.2	0.303	74.5 ± 6.7	80.0 ± 14.1	0.445
Hemoglobin, g/dl	12.6 ± 0.5	12.9 ± 1.0	12.4 ± 0.1	0.702	9.7 ± 1.5	9.0 ± 1.3	0.339
MCV, fl/cell	85.3 ± 6.1	88.3 ± 3.9	84.9 ± 1.8	0.221	74.5 ± 7.2	72.5 ± 13.3	0.739
MCH, pg/cell	27.6 ± 1.9	28.6 ± 1.7	27.7 ± 0.9	0.282	22.6 ± 3.0	21.3 ± 5.6	0.678
MCHC, g/dl	31.6 ± 1.0	32.2 ± 1.4	32.4 ± 1.5	0.541	30.1 ± 1.4	29.2 ± 2.4	0.739
RBC, million/mm^3^	4.5 ± 0.5	4.3 ± 1.0	3.5 ± 0.4	0.131	4.4 ± 0.6	4.3 ± 0.5	0.934
WBC, thousand cells/mm^3^	7.5 ± 1.8	9.2 ± 4.3	7.5 ± 0.6	0.823	8.3 ± 3.3	7.6 ± 1.6	0.739
Platelets, lakhs/mm^3^	2.7 ± 0.7	3.1 ± 1.2	2.4 ± 0.2	0.503	3.3 ± 0.9	3.6 ± 0.7	0.533
PCV, %	38.2 ± 3.1	35.5 ± 7.4	30.1 ± 4.3	0.079	32.3 ± 4.5	30.6 ± 2.1	0.360
RDW, %	16.7 ± 3.8	17.6 ± 6.2	20.6 ± 8.4	0.664	17.8 ± 3.5	22.8 ± 12.2	1.000
Urea, mg/dl	17.3 ± 5.2	15.6 ± 2.7	20.5 ± 5.0	0.344	17.3 ± 5.1	20.0 ± 5.7	0.558
Creatinine, mg/dl	0.7 ± 0.1	0.7 ± 0.1	0.7 ± 0.1	0.344	0.7 ± 0.1	0.7 ± 0.1	0.965
Ferritin, ng/ml	40.0 ± 19.0	107.2 ± 25.2	126.3 ± 28.6	0.001*	10.6 ± 8.6	28.5 ± 3.3	0.007*

Table 4 compares the distribution of fibroid subjects with iron deficiency anemia and those with iron deficiency state based on their vitamin D levels; 28 (93.3%) subjects were found to have vitamin D deficiency, and two (6.7%) had vitamin D insufficiency in the iron deficiency anemia group. Five subjects in the study were found to have an iron deficiency state and all of them had vitamin D deficiency.

**Table 4:** Distribution of fibroid subjects with iron deficiency anemia and subjects with iron deficiency state according to serum vitamin D levels

Vitamin D levels	Fibroid subjects with iron deficiency anemia, n (%)	Fibroid subjects with iron deficiency, n (%)
Vitamin D deficiency	28 (93.3)	5 (100)
Vitamin D insufficiency	2 (6.7)	0

The odds ratio with 95% CI between the serum vitamin D groups (vitamin D deficiency and vitamin D insufficiency) and serum ferritin groups (low and normal ferritin levels) is shown in Table 5. The odds ratio was found to be 9.6 (95% CI: 1.1-80.9, p=0.019). This indicates that low ferritin levels are associated with vitamin D deficiency in fibroid subjects.

**Table 5:** Odds ratio and 95% CI for low and normal ferritin groups according to serum vitamin D levels CI: confidence interval

Vitamin D levels	Ferritin of <12 ng/ml	Ferritin of >12 ng/ml	Odds ratio
Vitamin D deficiency	23	24	9.6 (95% CI: 1.1–80.9, p=0.019)
Vitamin D insufficiency	3	10	

**Figure 1:**
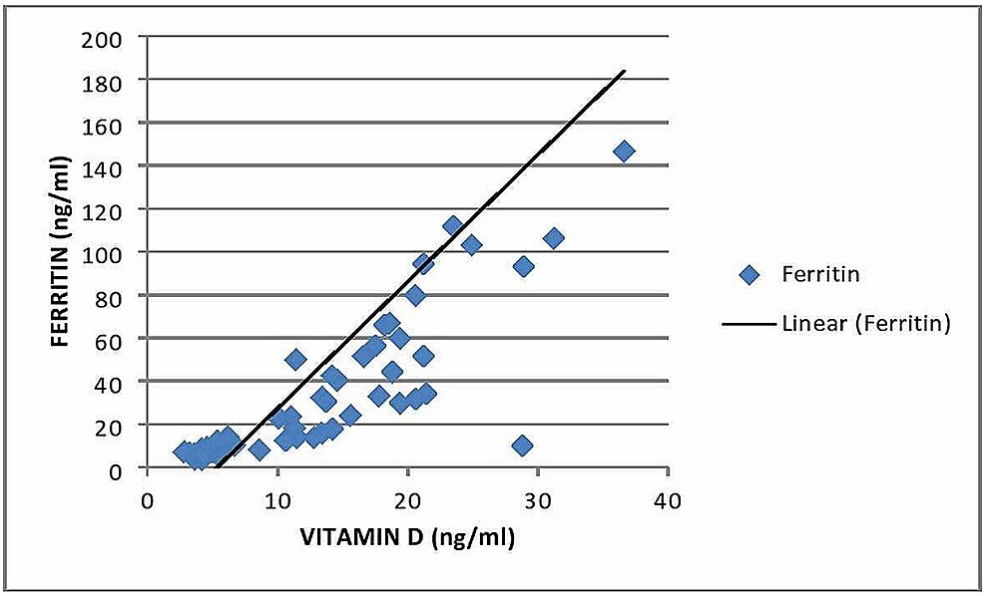
Scatter plot of serum ferritin against serum vitamin D levels Pearson correlation coefficient (r)=0.616 (p<0.001)

Figure 1 shows the Pearson correlation (r) between the serum ferritin levels and serum vitamin D levels among the study groups. A strong positive correlation of (r)=0.616 (p<0.001) was obtained.

## Discussion

Uterine fibroids (submucosal, intramural, and sub­serosal fibroids) have different effects; submucosal and intramural types are presumed to cause heavy menstrual bleeding leading to anemia [13]. Factors like estro­gen and progesterone, black ethnicity, and obesity are well-known risk factors for fibroids. Reproductive factors such as nulliparity, early menarche, and the use of oral contraceptives before the age of 16 years also contribute to its causation [14]. Hypovitaminosis D is believed to be a major risk factor for the development of uterine fibroids.

Recently, vitamin D has been found to have a protective effect, and both the initiation and growth of uterine fibroid might be inhibited with vitamin D supplementation [15]. Serum ferritin indicates the total body iron storage, and it represents iron status. Ferritin is decreased in subjects with iron deficiency anemia but may be increased in subjects with anemia of chronic disease and inflammation [16]. In the present study, most of the cases had microcytic hypochromic anemia; low levels of serum ferritin could be due to iron deficiency resulting from an inadequate intake of dietary iron or a negative iron balance. Katsumata et al. [17], in a rat model, have reported that iron deficiency decreased the levels of 1,25-dihydroxycholecalciferol and osteocalcin concentrations, suggesting that hydroxylation of vitamin D is dependent on iron, and hence iron-deficient rats had lower concentrations of the active form of vitamin D. In the present study, ferritin level and Hb level were the most significant factors affecting vitamin D levels. This result is consistent with previous studies [18,19]. Exposure to sunlight was more or less adequate in both the study groups, further suggesting the interrelationship of ferritin and vitamin D levels.

In terms of anemia, some studies have reported that vitamin D is positively associated with ferritin levels [20,21]. In the present study, vitamin D levels were reduced in patients with uterine fibroid and it was statistically significant in fibroid cases with anemia. Low serum vitamin D levels in fibroid cases could be due to the fact that vitamin D reduces the effect of transforming growth factor β3 (TGFβ3)-mediated expression of collagen type-1, and the expression and activity of metalloproteinase (MMP-2, MMP-9) that degrades the extracellular matrix [22]. Earlier studies have also reported the role of vitamin D in regulating the cellular signaling pathways associated with cell growth and proliferation, i.e., PCNA, CDK1, CDK2, CDK4, apoptosis, i.e., BCL2, BAD, caspase 3 as well as steroid hormone receptors. We found significantly low levels of vitamin D in fibroid cases with anemia. Serum ferritin levels are regulated by hepcidin, which plays a role in reducing iron absorption from the intestine. Vitamin D regulates the hepcidin-ferroportin axis in macrophages and the increase of vitamin D is known to reduce systemic hepcidin levels that ameliorate anemia [23]. Globally, various studies have been conducted to analyze the association between vitamin D and ferritin. Seong et al. [24] and Andıran et al. [25] reported that serum 25(OH)D was positively correlated with serum ferritin levels in patients in Korea and the US, respectively. However, Monlezun et al. [26] and de Castro et al. [27] reported that serum 25(OH)D was not associated with serum ferritin levels in adults from the US and Portugal, respectively. These inconsistent results may be due to the different populations, ethnic groups/countries, and the differences among the subjects of the studies (e.g., gender, absence or presence of diseases).

A strong positive correlation between serum ferritin and vitamin D levels was observed in the present study, which is in accordance with earlier findings. The odds ratios for iron deficiency and iron-deficiency anemia in subjects with vitamin D deficiency [25(OH)D of <15 ng/ml] were 1.86 (95% CI: 1.07-3.22) and 2.59 (95% CI: 1.11-6.07) in healthy Korean women as reported by Lee et al. [28]. In addition, Suh et al. [29] reported that 25(OH)D levels were lower in Korean women with iron deficiency anemia than in those without iron deficiency anemia (p<0.001). In our study, the finding of an odds ratio of 9.6 (1.1-80.9, p=0.019) with a 95% CI suggested that low ferritin levels are associated with vitamin D deficiency in fibroid uterus subjects. As reported, female gender, winter season, lack of sunlight exposure, residence in high latitudes, ethnicity (dark skin), clothing (clothing with low exposure), obesity, low socioeconomic status, and malnutrition are the factors affecting vitamin D levels. The present study found serum ferritin levels significantly affecting serum vitamin D levels in fibroid uterus cases with anemia.

## Conclusions

Fibroid uterus cases with microcytic hypochromic anemia were found more prone to have vitamin D deficiency in the population of the Eastern Zone of India as compared to fibroid uterus women presenting without anemia. Serum ferritin and Hb levels were the important factors affecting serum vitamin D values, as exposure to sunlight was more or less adequate in both the study groups.

A strong positive correlation was observed between serum ferritin and vitamin D levels, suggesting that fibroid uterus cases with microcytic hypochromic anemia should also be evaluated for vitamin D deficiency, as timely corrective measures would improve the treatment outcomes in women with fibroid uterus.
